# Identification of Autism Subtypes Based on Wavelet Coherence of BOLD FMRI Signals Using Convolutional Neural Network

**DOI:** 10.3390/s21165256

**Published:** 2021-08-04

**Authors:** Mohammed Isam Al-Hiyali, Norashikin Yahya, Ibrahima Faye, Ahmed Faeq Hussein

**Affiliations:** 1 Centre for Intelligent Signal and Imaging Research (CISIR), Department of Electrical and Electronic Engineering, Universiti Teknologi PETRONAS, Seri Iskandar 32610, Perak, Malaysia; mohdisam_19001725@utp.edu.my (M.I.A.-H.); ibrahima_faye@utp.edu.my (I.F.); 2 Biomedical Engineering Department, Faculty of Engineering, Al-Nahrain University, Baghdad 10072, Iraq; ahmed.f.h.1976@gmail.com

**Keywords:** autism spectrum disorder, multi-class classification, resting state fMRI, BOLD signal, scalogram

## Abstract

The functional connectivity (FC) patterns of resting-state functional magnetic resonance imaging (rs-fMRI) play an essential role in the development of autism spectrum disorders (ASD) classification models. There are available methods in literature that have used FC patterns as inputs for binary classification models, but the results barely reach an accuracy of 80%. Additionally, the generalizability across multiple sites of the models has not been investigated. Due to the lack of ASD subtypes identification model, the multi-class classification is proposed in the present study. This study aims to develop automated identification of autism spectrum disorder (ASD) subtypes using convolutional neural networks (CNN) using dynamic FC as its inputs. The rs-fMRI dataset used in this study consists of 144 individuals from 8 independent sites, labeled based on three ASD subtypes, namely autistic disorder (ASD), Asperger’s disorder (APD), and pervasive developmental disorder not otherwise specified (PDD-NOS). The blood-oxygen-level-dependent (BOLD) signals from 116 brain nodes of automated anatomical labeling (AAL) atlas are used, where the top-ranked node is determined based on one-way analysis of variance (ANOVA) of the power spectral density (PSD) values. Based on the statistical analysis of the PSD values of 3-level ASD and normal control (NC), putamen_R is obtained as the top-ranked node and used for the wavelet coherence computation. With good resolution in time and frequency domain, scalograms of wavelet coherence between the top-ranked node and the rest of the nodes are used as dynamic FC feature input to the convolutional neural networks (CNN). The dynamic FC patterns of wavelet coherence scalogram represent phase synchronization between the pairs of BOLD signals. Classification algorithms are developed using CNN and the wavelet coherence scalograms for binary and multi-class identification were trained and tested using cross-validation and leave-one-out techniques. Results of binary classification (ASD vs. NC) and multi-class classification (ASD vs. APD vs. PDD-NOS vs. NC) yielded, respectively, 89.8% accuracy and 82.1% macro-average accuracy, respectively. Findings from this study have illustrated the good potential of wavelet coherence technique in representing dynamic FC between brain nodes and open possibilities for its application in computer aided diagnosis of other neuropsychiatric disorders, such as depression or schizophrenia.

## 1. Introduction

Autism spectrum disorder (ASD) is a psychiatric disorder caused by impairment in brain functions [1]. ASD patients suffer from weakness in verbal and non-verbal communication and difficulty in social activities, which may influence their life quality and interpersonal skills. A report by the World Health Organization has indicated that, in 2019 alone, 1 in 160 children has ASD [2]. One of the challenges in clinical diagnosis of ASD is the lack of objective interpretation mechanisms of ASD  [3]. Current practise of clinical diagnosis of ASD is based on behavioral assessment, but with high heterogeneous nature of ASD and varying clinical symptoms [4] may render the diagnosis to be inaccurate. Based on the Diagnostic and Statistical Manual of Mental Disorders (DSM-4), ASD is categorized into three subtypes based on symptom variations; autistic disorder (ASD), Asperger’s disorder (APD), and pervasive developmental disorder not otherwise specified (PDD-NOS) [5]. However, accurate behavioral assessment requires a trained psychiatrist and is susceptible to human error either during the assessment or interpreting the results. This issue may hinder the treating progress of the ASD patients. Indeed, an objective early ASD detection and suitable therapeutic plans choice are essential in improving the condition and quality of life of the ASD patients. In the past two decades, neuroscience studies have been making progress in characterizing biomarkers for interpreting neural mechanisms of ASD using functional brain imaging modalities [6]. In a similar trend, there is also a rapid increase in application of artificial intelligence (AI) models in the medical diagnosis field, especially in psychiatric disorders [7]. The use of AI has improved the diagnosis results and decreased the decision time associated with the traditional diagnosis method. In ASD cases, several studies are devoted to using resting-state functional magnetic resonance imaging (rs-fMRI) data with different types of AI classifiers [8]. Generally, the functional magnetic resonance imaging (fMRI) is a non-invasive modality and has emerged as a powerful tool for depicting brain functionality of the cortex to deep brain regions. The fMRI provide the estimation of neuronal activity based on blood-oxygen-level-dependent (BOLD) [9], as indirect signals that reflect the fluctuation in brain blood flow and blood oxygenation levels coupled to underlying neuronal activity [10]. In a resting state experiment, the functional brain networks are detected without any specific tasks [11]. Using the BOLD signals analysis at resting state helps neuroscientists to understand the fundamental mechanism of brain functioning of ASD patients [7]. One common measure of brain functionality is by using functional connectivity (FC) of BOLD signals between brain regions which gives indication of the statistical correlation between different regions [12]. In general, there are two main models applied in BOLD signals analysis, static (SFC) and dynamic (DFC) functional connectivity [13], both can be used for detection of psychiatric disorders [14]. The SFC and DFC differ in their method of calculating the correlation coefficients. The SFC represents the interaction between pairs of brain nodes as a single correlation coefficient calculated from the BOLD signals of the entire scan but no temporal variations are considered in the calculation. In contrast, the DFC is calculated using wavelet transform, hence capturing both time and frequency details of the BOLD signals. It indicated the coherence strength between pairs of brain regions, represented in the form of two-dimensional matrix called scalogram.

### Classification of ASD Using Functional Connectivity (FC)—Related Works

Many researches on brain FC are focussing on identifying the neurological biomarkers for ASD patients [15]. Application of SFC [10,12,16,17,18], and DFC [19] for detection of ASD in rs-fMRI has been investigated in the past papers. This section summarized the related works on ASD classification algorithms based on SFC and DFC as inputs to machine learning (ML) [16,17,19] or deep learning architecture [10,12]. Recent advancement in deep learning has enables the transfer learning technique which is known to effectively improve the identification accuracy of diagnostic algorithms [20,21]. The number of SFC features generated from correlation coefficients of the BOLD signals usually amount to the order of thousands but the classification accuracy based on these features still need to be improved. This is because only some regions of the brain carry the informative features that discriminate ASD vs. normal control (NC). In [16] Chen et al. used Pearson correlation of pairwise BOLD signals in low-frequency bands as input to support vector machine (SVM), achieving 79% accuracy in ASD vs. NC prediction. In another work by Abraham et al. [17], covariance matrices of pairwise BOLD signals are used as the input features to an SVM classifier giving 67% accuracy. Recently, Chaitra et al. [18] achieved 70.1% accuracy for ASD prediction using combination of Pearson correlation with complex brain network measurements as input features to the recursive-cluster-elimination-SVM (RCE-SVM) algorithm.

Apart from using conventional ML techniques, deep learning (DL) algorithms are also used in the development of binary classification algorithms of ASD v. NC using SFC features. The recent one by Heinsfeld, et al. [12], used two stacked denoising autoencoders to transfer 19,900 features of FC extracted based on the Pearson correlation into the deep neural network (DNN), giving 70% classification accuracy. The other one is by Zeinab, et al. [10], where Pearson correlation coefficients are input to the CNN as images and binary classification accuracy of 70.2%. An approach using DFC between pairwise BOLD signals by employing wavelet coherence transforms (WCT) was proposed by Bernas et al. [19]. The WCT coefficients are used as the input vector to SVM achieving 80% accuracy for ASD vs. NC prediction. Apart from FC, the time-frequency components of BOLD signals are represented into 2D images in our previous study [21] and used as input to the CNN models for feature extraction, and k-nearest neighbors (KNN) as a best classifier algorithm with 85.9% accuracy.

The ML-based techniques using either SFC or DFC are not able to capture the topological information within the brain regions and the relationships between the neural activity features and the clinical symptoms [22]. This is proven to be more difficult especially on the highly heterogeneous symptoms, such as ASD subtypes. However, the results of the studies mentioned above have had a minimal clinical impact. The reason for that is the vast majority of these studies have typically reported differences between ASD patients and normal controls, with best accuracy of 80%. At the same time, in clinical decisions, the ASD subtypes should not be ignored. Thus, the multi-class classification algorithm is critical in assisting ASD health practitioners in correct diagnosis of ASD subtypes. It is to be noted that the SFC features may not carry sufficient information for multi-class classification [23]. Hence, a better choice would be using the DFC which represents correlation as a function of time-frequency between BOLD signals. There is evidence that DFC patterns may play a crucial role in identifying subtypes of psychiatric disorders, such as ASD. Indeed, the DFC patterns have been rarely investigated as input features for ASD classification models. In [19] wavelet coherence transforms (WCT) and SVM are used for binary classification of ASD. With accuracy of 80%, this leaves much room for improvement.

In this work, we developed an ASD classification algorithm based on wavelet coherence of BOLD signals and CNN. In specific, the calculation of the wavelet coherence are calculated between the top-ranked brain node to the rest of the nodes of automated anatomical labeling (AAL) atlas. Method of statistical significance analysis is employed on the power spectral density (PSD) of the BOLD signals from 116 brain nodes to determine the most significant node in multi-class (3-level ASD and NC) settings. A total of 115 wavelet coherence scalograms generated for each subject, represent the time-frequency resolution of the signal which may provide valuable information in identification of ASD subtypes. Results generated from this work are using dataset from the autism brain imaging data exchange (ABIDE) [24] which is an online data source for rs-fMRI data of ASD patients and normal control (NC) groups collected from several neuroscience laboratories worldwide. The rest of this study is organized as follows. Section 2 describes the materials and proposed methods, including the data preparation, BOLD-dynamic features extraction and classification models. The results and comparison with benchmark studies are explained in Section 3 providing conclusions and future works in this area in Section 4. The objective of this study is to develop an automated ASD subtypes classification using DFC patterns of rs-fMRI data. DFC features extracted using pairwise WCT, inherently leveraging the rich information of the WCT both in time and frequency domains.

## 2. Materials and Methods

**Figure 1:**
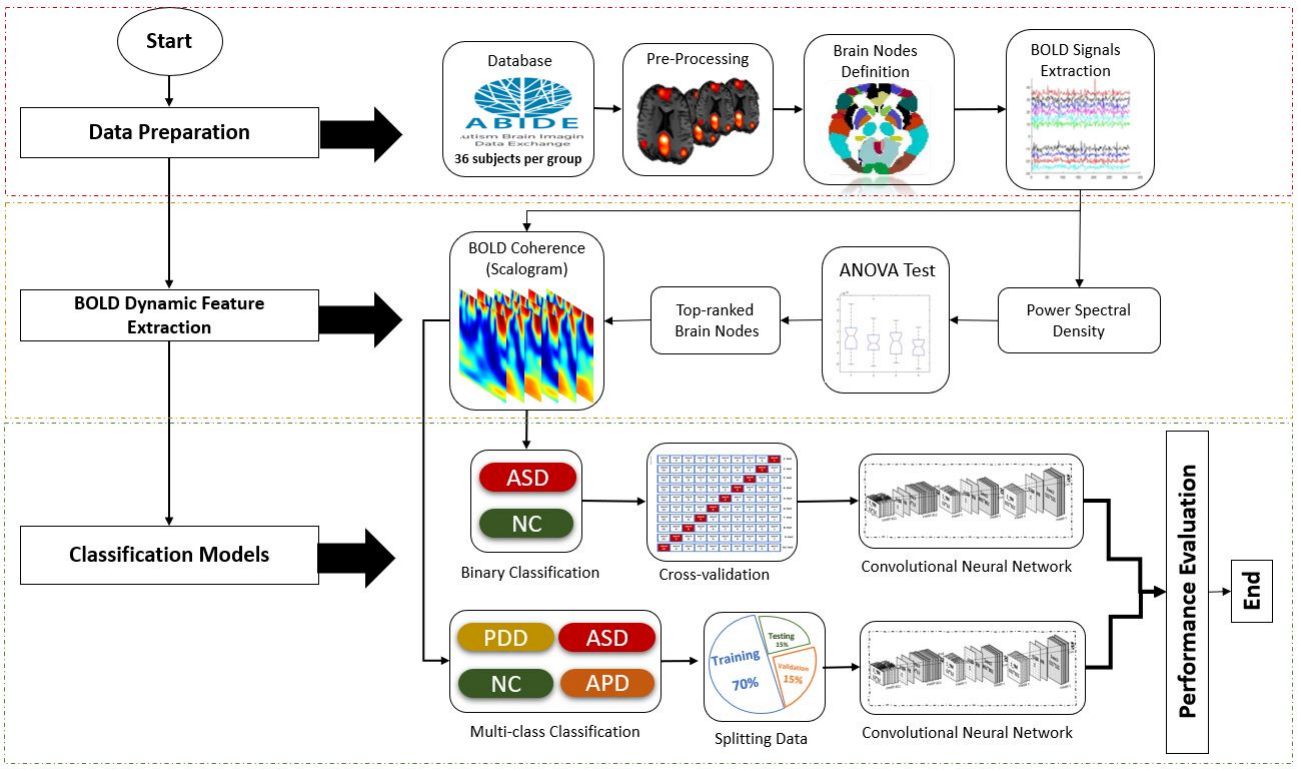
Methods for development of binary and multi-class classification of BOLD fMRI signals using wavelet coherence and CNN.

Overall methodology in the development of automated ASD subtypes classification using DFC patterns of rs-fMRI data is illustrated in Figure 1. Here, we consider 2 classification techniques, binary and multi-class classifications.

### 2.1. Data Preparation

In this study, resting-state fMRI data are collected from multiple sites of ABIDE dataset [24]. The ABIDE data contain longitudinal relaxation time (T1) structural MRI brain images, fMRI images, and phenotypic information of the patients. Although ABIDE has more than 1000 subjects, with 446 ASD and 590 NC, coming from various contributors, not all ASD data are labeled based on the subtypes of DSM-4. Specifically, the available data based on DSM-4 are 323 ASD, 87 APD, and 36 PDD-NOS subjects. To avoid the issue of an imbalanced dataset which might affect the performance of the classifier, the number of subjects for each group is set at 36, the smallest sample size of ASD class. Details on the dataset, its scanning parameter and the number of subjects are listed in Table 1. All datasets were acquired using 3 Tesla (3T) MRI scanners.

The use of multi-site data introduces larger data variance during the training of the classifier due to differences in scanning parameters or type of scanner. The multi-site data may pose a challenge in generalizing the trained ASD classifiers [12] and this issue will be experimented here using leave-one-site out validation method.

The selected data were pre-processed by using the DPARSF Matlab toolbox followed by BOLD signals extraction [25] from 116 regions of the automated anatomical labeling (AAL) atlas. The AAL atlas divides the brain region into 116 nodes, as shown in Table A1 under the Appendix A. Since there is variation in recording time, the number for sample points of the BOLD signals varies from one site to the other. Therefore, in order to work with the same length of data, the signal is truncated to the shortest sample point, which is 145 time points.

### 2.2. Statistical Analysis Using Power Spectral Density (PSD)

The dimension of the BOLD time series for each subject is 145-points × 116-region. If the WCT between all brain nodes are to be used in this investigation, the number of scalogram images for each subject alone will be (115×116)/2=6670 which is a large number of images. Furthermore, some of these images may not have a meaningful contribution in the classification of ASD subtypes, thus, would be detrimental to the classification performance. Therefore, a group-level statistical test is performed to select the most significant brain node based on the PSD of the BOLD signals. Power spectral density of the BOLD time-series signals is estimated using Welch method [26].

Detail of the steps for finding the top-ranked node using the mean value of PSD is given in Algorithm 1. The PSD values of each 116 brain regions determined using Welch are normalized to zero mean and standard deviation of 1. Normalization is deemed necessary here since the dataset is obtained from different sites, thus ensuring the reliability of the statistical analysis. Next, the average of the normalized PSD values are used as the input for one-way analysis of variance (ANOVA) test.
**Algorithm 1:** Method of finding the top-ranked node in discriminating 3-level ASD subtypes and NC using mean value of PSD.1.Input data = matrix (m×n)*m* = 36 (number of subjects), *n* = 4 (number of group)2.BOLD signals = $t\times R_{j}$*t* = time points, $R_{j}$ = node number (*j* = 1, 2, …, 116)3.PSD = estimate PSD for each BOLD signal4.$PSD_{n}$ = normalize and determine the average of the PSD values5.$G_{j}$ = cluster the outcome of (4) into matrix (m×n)6.*p*-value = run ANOVA test for each cluster ($G_{j}$)While j ≤ 116 repeat step 6If *p*-value ≤ 0.05save *p*-value at Telse T = empty7.F = Find top-ranked node based on T-index

### 2.3. Wavelet Coherence of BOLD Time-Series Signals

Wavelet coherence of two signals is a measure of linear interaction or correlation between the signals. Since the wavelet transform provides both time and frequency domain representation of signals, WCT measures the mean resultant vector length of the cross-spectral density between two signals. In another word, the WCT will provide the phase synchronization between the pairwise BOLD signals [13,27].

Firstly, the time-frequency components for each BOLD signal were extracted by using a continuous wavelet transform (CWT). The CWT coefficient is defined as the convolution of the BOLD time series x(t) with the scaled and translated version of the mother wavelet $\psi_{a,b}\left(t\right)$ [28], as shown in Equation (1).

(1) $$CWT\left(a,b\right)=\frac{1}{\sqrt{a}}\int_{-\infty }^{\infty }x\left(t\right)\psi^{*}\left(\frac{t-b}{a}\right)dt,$$

where *a* denotes wavelet scale, *b* denotes positions and * denotes the complex conjugate [29]. The complex Morlet wavelet was selected as the mother wavelet. Morlet has the best ratio (1.03) between frequency band and wavelet scale, which helps interpreting results in the frequency domain [28]. CWT is becoming a popular method in biosignal analysis due to its ability to uncover meaningful information of non-stationary signals such as electroencephalogram (EEG) [30] and BOLD fMRI signals [19,21]. In fact, WCT based on CWT, characterizes coherence measures between two signals at multiple time scales, essentially makes no assumption about the stationarity of the input signals. Accordingly, CWT has achieved reasonable trade-off between time and frequency components [31,32].

In the subsequent step, the common power between the pairwise of BOLD signals *x*, *y* is measured at various scales *a* and time shift *b* by Equation (2):

(2) $$C_{xy}\left(a,b\right)=S\left(C_{x}^{*}\left(a,b\right)C_{y}\left(a,b\right)\right)$$

where $C_{x}\left(a,b\right)$ and $C_{y}\left(a,b\right)$ denote the CWT of *x* and *y* at scales *a* and positions *b*, the superscript * is the complex conjugate, and *S* is a smoothing operator in time and scale.

Then, the WCT between *x* and *y* is calculated by Equation (3):

(3) $$WCT_{xy}=\frac{|C_{xy}\left(a,b\right){|}^{2}}{\left(S|C_{x}\left(a,b\right){|}^{2}\right)\left(S|C_{y}\left(a,b\right){|}^{2}\right)}$$

The WCT coefficients were represented as 2-D images involving the phase synchronization features of pairwise BOLD signals called scalogram images and will be used as the input of CNN for classification.

The scalogram image is a form of DFC between 2 BOLD signals, represented as phase synchronization patterns. In our proposed study, all coherent synchronicity features are represented as 224 × 224-pixel images and used as the input for CNN in binary and multi-class ASD classification models.

**Figure 2:**
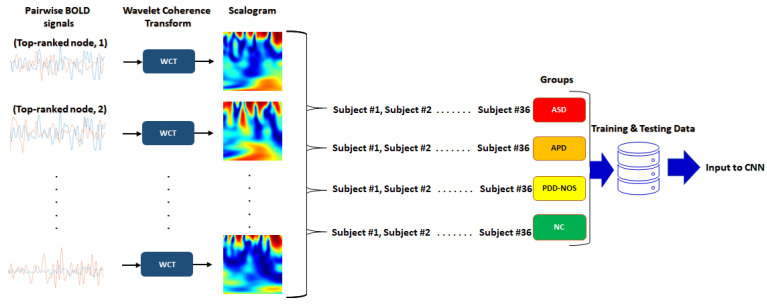
Wavelet coherence of pairwise BOLD signals between top-ranked node and 115 brain nodes.

These images are the WCT between the most significant node, as determined using ANOVA test with the rest of 115 brain regions. This pairwise calculation of WCT between the top-ranked node and 115 brain regions is illustrated in Figure 2, which will produce a total of 115 scalograms per subject.

### 2.4. Convolutional Neural Network (CNN)

**Figure 3:**
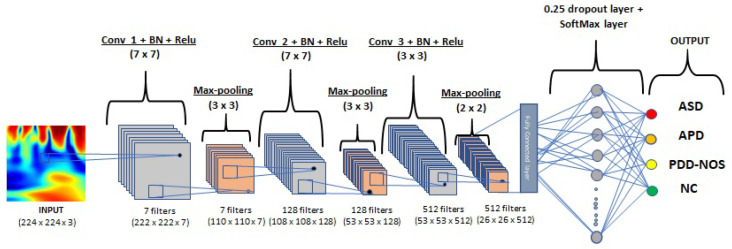
Training parameters: Batch size = 32, Epochs = 20, Learning rate = 0.0005. 3-layer CNN architecture for wavelet coherence scalogram classification into three ASD subtypes and normal control.

CNN is one of the essential deep neural networks related to applying local convolution filters for extracting regional information. CNNs are designed to process multiple data types, particularly two-dimensional variables, and are specifically influenced by the working principle of the brain’s visual cortex. There is a hierarchy of two basic cell types in the visual cortex: plain cells and complex cells. Simple cells respond to primitive patterns in visual stimulation sub-regions, and complex cells synthesize information from simple cells to recognize more complicated types. Since the visual cortex is such an efficient and normal visual processing device, CNNs are used to mimic three main ideas: local connectivity, position invariance, and local transformation invariance. Groups of local weighted sums, called feature maps, are obtained at the end convolution layer by computing convolutions between local patches and weight vectors called filters for extracting the strongly clustered sub-regions of features. In addition, because similar patterns may occur irrespective of the data position, filters are repeatedly implemented throughout the whole dataset, which often increases the accuracy of the trained network by minimizing the amount of parameters to be trained [33]. In this work, We proposed a 3-layer CNN model for identifying ASD subtypes based on scalogram classification, using the CNN structure as shown in Figure 3.

### 2.5. Performance Evaluation Metric

In order to analyze the performance of the proposed models, the following metrics (4) to (8) were chosen. True positive (TP) is the number of ASD patients, and true negative (TN) is the number of NC individuals correctly identified. Conversely, false positive (FP) is the number of ASD patients, and false negative (FN) is the number of NC individuals incorrectly identified.

(4) $$\mathrm{Precision}=\frac{TP}{TP+FP}$$

(5) $$\mathrm{Sensitivity}=\frac{TP}{TP+FN}$$

(6) $$\mathrm{Specificity}=\frac{TN}{TN+FP}$$

(7) $$\mathrm{Accuracy}=\frac{TP+TN}{TP+TN+FP+FN}$$

(8) $$F-\mathrm{score}=2\times \frac{Precision\times Sensitivity}{Precision+Sensitivity}$$

The sensitivity measures the effectiveness of proposed models to identify ASD patients correctly, and the specificity measures the effectiveness of models to identify NC individuals. Accuracy is the percentage of total effectiveness of a model. To evaluate our proposed models practically like in clinical set up, we calculate precision and F-score, respectively. The precision refers to the percentage of compatibility between the actual ASD patient class and patient class identified by the model. F-score is calculated from the precision and sensitivity of the model. The highest possible value of an F-score is indicating a perfect model performance.

Moreover, to analyze the general classification performance of multi-class models, we have chosen the macro-average evaluation, which makes an averaging calculation by class, not the subjects. The macro-average reduces the multi-class identifications down to multiple sets of binary classification, calculates the corresponding metric for each of the binary cases, and then averages the results [34].

## 3. Results and Discussion

In this section, the performance of ASD classification algorithms using wavelet coherence of rs-fMRI signals and CNN are evaluated. Two frameworks are experimented, binary classification (ASD and NC) and multi-class classification (ASD, APD, PDD-NOS, and NC). Prior to the classification, the most significant brain nodes need to be determined to ensure meaningful wavelet coherence features are input to the CNN.

### 3.1. Selection of Top-Ranked Brain Node for Classification of ASD Subtypes via Statistical Analysis

**Figure 4:**
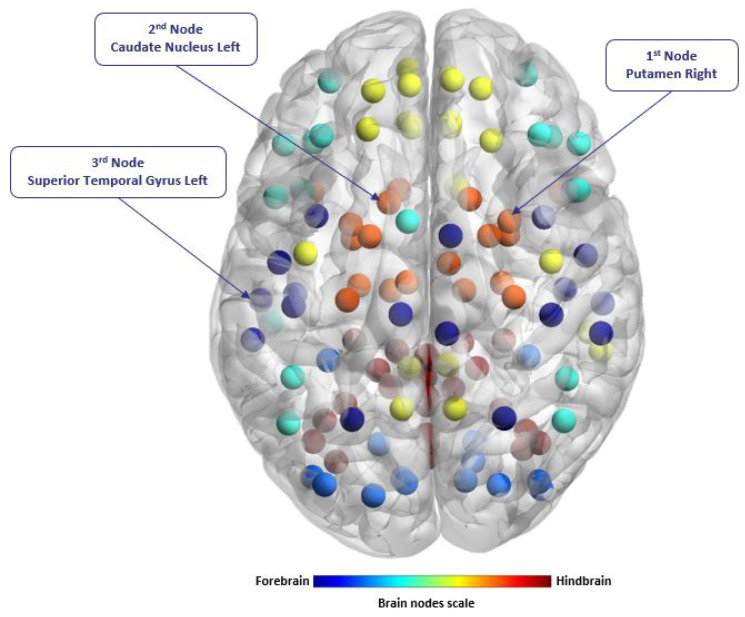
Top-three ranked brain nodes for classification of 3-level ASD subtypes and NC, determined using ANOVA analysis of mean value of PDC.

**Figure 5:**
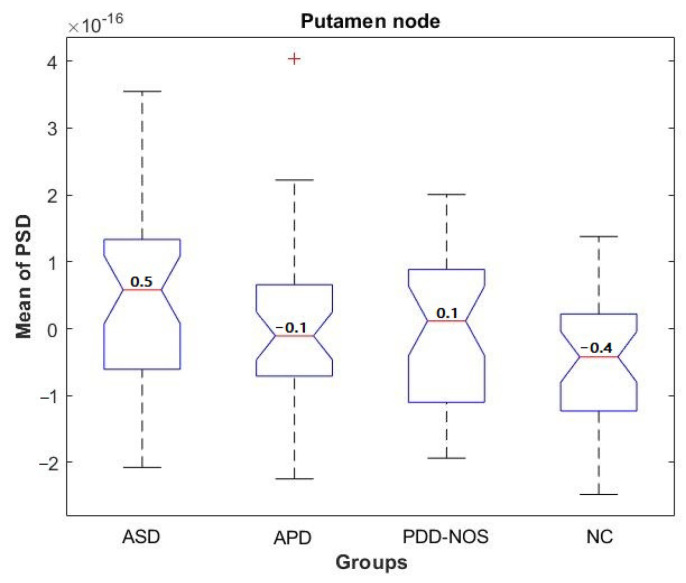
Statistical comparison of 3 ASD subtypes and NC based on PSD of putamen nodes.

As the first step in a statistical significance test, the mean PSD values of BOLD signals from 116 brain regions are determined as the input to group-level statistical significance tests. Results of the *p*-value are tabulated in Table A1, under Appendix A. From the *p*-value, it can be revealed that putamen_R node is the most significant node in discriminating the 3 ASD classes and NC. The location of putamen_R node, the 2nd (caudate nucleus_L) and 3rd ranked nodes (superior temporal gyrus_L), are shown in Figure 4. Further analysis on the PSD values of putamen_R node is shown as a boxplot in Figure 5. From the boxplot, it is clear that the PSD value is the highest for ASD among the 4 groups, while the lowest is for NC. These results indicate that there is significant variation of PSD based on putamen_R activity.

In other words, it is indicative that the top-ranked node activity plays an essential role in ASD subtypes classification. The results in this section corroborate the findings in neuroscience studies which reveal that the putamen and caudate are part of the basal ganglia group primarily responsible for motor learning, executive functions, behaviors, and emotions. Several neuroscience studies [1,35,36] demonstrated that the volume in the brain region of putamen node increase in the patients with ASD, followed by the volume differences in the brain region of putamen node among ASD subtypes might reflect the variations of the symptoms of ASD.

### 3.2. Binary Classification Using Wavelet Coherence of Top Three Significant Nodes

In the first experiment, we evaluated the significance of wavelet coherence features extracted from top-three nodes; putamen_R, caudate nucleus_L, superior temporal gyrus_L, and their combinations. Evaluation is conducted for discriminating ASD from NC using the proposed 3-layer CNN with the following training parameters: batch size = 32, epochs = 20, learning rate = 0.0005, adaptive moment estimation (ADAM) optimizer and ratio of training:validation:testing = 0.7:0.15:0.15.

The results for this experiment are presented in Table 2 where for single-node cases, the best accuracy is 89.2% by the top-ranked node putamen_R which is consistent with the result from ANOVA test, as presented in Section 3.1. As expected, the accuracy values for the 2nd and 3rd ranked nodes are both lower than the 1st node.

In the case of combined nodes, although more images are available for training and testing the CNN, the results show that it cannot exceed the performance of the 1st-node. Notably, 1st + 2nd nodes yielded the highest accuracy higher than 2nd and 3rd nodes alone but still lower than the 1st node alone. Subsequent combinations of 1st + 3rd nodes and 1st + 2nd + 3rd nodes still produce lower accuracy than the 1st node. Additionally, despite larger training images for combined nodes, the additional images do not carry meaningful features for discriminating ASD from normal NC

### 3.3. Binary Classification Using Wavelet Coherence of Putamen_R Node

In this section, the Putamen_R node will be used for classification of ASD from NC. Except for the cross-validation (CV) framework, the CNN training parameters are the same as in the previous section. The training of the CNN is tested using 3 optimizers, root mean square propagation (RMSPROP), stochastic gradient descent with momentum (SGDM), and adaptive moment estimation (ADAM), and the results for different values of folds are presented in Table 3. From the values of accuracy, sensitivity, specificity, precision, and F-score, it is evident that ADAM optimizer results in the best result for k-fold CV, thus, the subsequent experiments are conducted using ADAM optimizer. Evaluation of the proposed algorithm is further tested using k-fold cross validation and the result is tabulated in Table 4. As expected, the performance improved as the number of fold increases. However, it is notable that only marginal improvement is achieved as the fold number is increased from 10 to 15 and 20.

Since the rs-fMRI data were aggregated across ABIDE’s multi-site, validation technique based on leave one-site-out is used to investigate how well the CNN model generalized over different datasets. The results of this experiment are presented in Table 5. With average accuracy of 86.8±0.7% the proposed binary classification model is considered as robust against the effects of different types of MRI scanners and scanning parameters, as listed in Table 1.

Further performance comparison for binary classification of ASD vs. NC with other related work is presented in Table 6. Methods based on static FC of Pearson correlation [10,12,17] and of covariance matrix [16] can only yield the highest accuracy of 79.2%, inferior to the dynamic FC. Our proposed method that inputs WCT of putamen_R and 115 brain regions to CNN has shown to result in a good accuracy of 89.8%, that is 9.8% higher than the dynamic FC based method proposed by Bernas et al. [19]. Although Bernas et al. [19] used the same WCT, the calculation of the WCT is between 7 brain networks and only in-phase components are input to the SVM classifier.

### 3.4. Multi-Class Classification

**Figure 6:**
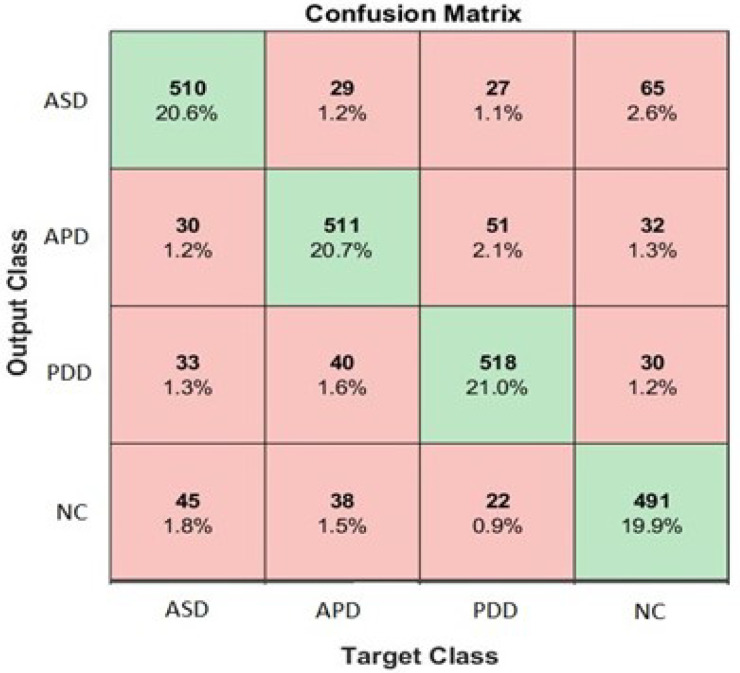
Confusion matrix for classification of WCT images of ASD subtypes and NC using ADAM optimizer.

In the last experiment, the proposed model is trained for multi-class classification of 3-level ASD and NC taking scalogram images for four groups as its input. To evaluate the performance of the proposed CNN model, the scalogram data is divided into 0.7 as training dataset, 0.15 as validation dataset and 0.15 as testing datasets. The rest of the training parameters are the same as in Section 3.2. The proposed CNN is trained using ADAM, SGDM, and RMSPROP optimizer and the results for each optimizer are presented in Table 7. The best performance is achieved by the proposed CNN with ADAM optimizer giving macro-average accuracy 82.1%. Detailed performance of each class is presented in the confusion matrix, as shown in Figure 6. It is worth highlighting that to date, the ASD subtypes classification has not been done in literature so in this first attempt, with macro-average accuracy 82.1%, there are still opportunities for further enhancements to the classification algorithm.

## 4. Conclusions and Future Works

In this study, we proposed scalogram-based classification models using the CNN to identify ASD subtypes. The scalogram is generated based on wavelet coherence of pairwise rs-fMRI BOLD signals of top-rank node and the rest of 115 brain nodes. The multi-class datasets of ASD subtypes comprising 144 subjects are downloaded from the multi-site of ABIDE website. Using statistical significance analysis of mean PSD values, putamen_R node is identified as the most significant node. The WCT scalograms of putamen_R and the rest of 115 nodes are then used as the input for training and testing the 3-layer CNN model. In general, the WCT of pairwise BOLD signals is a 2D feature representation that measures the phase synchronization between putamen_R to other brain nodes. Clearly, the extracted feature is proven to be a discriminative BOLD signals descriptor for ASD subtypes and may be a potential biomarker for diagnosis of ASD. The accuracy of 89.8% for binary and 82.1% for multi-class classification, are obtained based on BOLD signals combined from all subjects in respective class, which may not give true measure of its performance for subject-based diagnosis. Therefore, training and testing the CNN on subject-based needs to be investigated for assessment of its diagnostic ability as in clinical practices. In addition, further investigation to improve its performance may consider utilizing different brain atlases, such as Craddock (CC200, CC400) that extract more information from the BOLD signals. Besides, the scalogram images can also be trained and tested on other CNN architecture, such as residual or inception blocks for better classification of ASD subtypes. Lastly, the phase synchronization between one significant brain node to the rest may also be applied for classification of other neuropsychiatric disorders such as ADHD, bipolar disorders, and schizophrenia. The present study approach of using WCT as DFC of rs-fMRI BOLD signals opens a possibility for further research on new biomarkers of psychiatric disorders.

## Figures and Tables

**Table 1:** Details fMRI ASD subtypes and NC dataset from ABIDE database, acquired using 3T MRI scanner.

Site	Country	Vendor	Voxel Size (mm${}^{3}$)	Flip Angle (deg)	TR (sec)	Time Points (sec)	Subjects				Total-per Site
							ASD	APD	PDD-NOS	NC	
NYU	USA	Siemens	1.3	7	2	175	9	8	5	9	31
SBL	Netherlands	Philips	1	8	2.2	195	9	5	6	9	29
SDSU	USA	GE	1	4.5	2	175	9	6	2	9	26
Trinity	Ireland	Philips	1	8	2	145	-	4	7	-	11
Yale	USA	Siemens	1	9	2	195	9	5	14	9	37
USM	USA	Siemens	1	9	2	235	-	-	1	-	1
KKI	USA	Philips	1	8	2.5	151	-	8	-	-	8
UM1	USA	GE	1.2	15	2	295	-	-	1	-	1
**Total**							36	36	36	36	144

Legend: NYU: New York University, SBL: Social Brain lab, SDSU: San Diego State University, Trinity: Trinity College Institute of Neuroscience, Yale: Yale School of Medicine, USM: University of Utah School of Medicine, KKI: Kennedy Krieger Institute, UM: University of Michigan, TR:Repetition Time.

**Table 2:** Performance of proposed CNN for binary classification using WCT of significant node(s) as the input images, where the number of subjects is ASD = NC = 36.

Node for WCT	Number of WCT Images per Class	Accuracy (%)
1st-node	4140	89.2
2nd-node	4140	84.9
3rd-node	4140	83.1
1st + 2nd-nodes	8280	85.5
1st + 3rd-nodes	8280	84.7
1st + 2nd + 3rd-nodes	12,420	81.7

**Table 3:** Percentage of accuracy, sensitivity, specificity, precision, and F-score (±standard deviation) of 10-folds cross-validation for binary classification.

Optimizer	Accuracy	Sensitivity	Specificity	Precision	F-Score
RMSPROP	84.5 ± 1.8	85.1 ± 2.5	84.3 ± 2.2	84.2 ± 2.8	84.6 ± 1.9
SGDM	87.2 ± 0.9	87.1 ± 1.4	87.4 ± 1.5	87.4 ± 1.5	87.2 ± 0.9
ADAM	89.2 ± 0.7	89.1 ± 2.5	89.5 ± 1.9	89.5 ± 2.5	89.2 ± 0.5

**Table 4:** Percentage of accuracy, sensitivity, specificity, precision, and F-score (±standard deviation) for binary classification of ASD vs. NC using k-fold cross-validation.

k-Folds	Accuracy	Sensitivity	Specificity	Precision	F-Score
5-fold	88.6 ± 1.5	88.7 ± 2.3	88.7 ± 2.3	88.6 ± 2.6	88.6 ± 1.5
10-fold	89.2 ± 0.7	89.1 ± 2.5	89.5 ± 1.9	89.5 ± 2.5	89.2 ± 0.5
15-fold	89.6 ± 1.6	88.9 ± 2.4	90.5 ± 1.8	90.6 ± 2.1	89.7 ± 1.5
20-fold	89.8 ± 1.7	90.1 ± 2.6	89.7 ± 2.2	89.6 ± 2.5	89.8 ± 1.7

**Table 5:** Percentage of accuracy, sensitivity and specificity (in%) for binary classification, ASD vs. NC using leave-one site validation.

Site	Accuracy	Sensitivity	Specificity	Precision	F-Score
NYU	87.5	88.3	86.8	86.5	87.4
SBL	86.9	87.6	86.2	85.9	86.7
SDSU	86.9	88.4	85.4	84.8	86.5
Yale	85.8	85.4	86.2	86.3	85.8
Mean	86.8	87.4	86.1	85.9	86.6

**Table 6:** Comparison of the proposed ASD binary classification with previous papers.

Paper	Classifier	FC Modelling	Method	Subject	Accuracy (%)
Chen et al. 2016 [16]	SVM	Static FC	Pearson correlation	240	79.2
Abraham et al. 2017 [17]	SVM	Static FC	Covariance matrix	871	67
Heinsfeld et al. 2018 [12]	DNN	Static FC	Pearson correlation	1035	70
Bernas et al. 2018 [19]	SVM	Dynamic FC	Wavelet coherence	54	80
Sherkatghanad et al. 2020 [10]	DNN	Static FC	Pearson correlation	871	70.2
Our proposed method	CNN	Dynamic FC	Wavelet coherence	72	89.8

**Table 7:** Macro-accuracy (in %) of multi-class classification using three optimization methods.

Optimizer	F1-Score(%)			Accuracy (%)
	ASD	APD	PDD-NOS	Overall
RMSPROP	79.6	80.7	81.7	80.2
SGDM	80.9	79.8	80.6	80.3
ADAM	81.7	82.3	83.6	82.1

## Data Availability

Not applicable.

## Appendix A

**Table A1:** *p*-Value for all brain nodes based on ANOVA test.

No.	Region Label	*p*-Value	No.	Region Label	*p*-Value	No.	Region Label	*p*-Value	No.	Region Label	*p*-Value
1	Precentral_L	0.191	31	Cingulum_Ant_L	0.485	61	Parietal_Inf_L	0.971	91	Cerebelum_Crus1_L	0.570
2	Precentral_R	0.115	32	Cingulum_Ant_R	0.911	62	Parietal_Inf_R	0.862	92	Cerebelum_Crus1_R	0.862
3	Frontal_Sup_L	0.061	33	Cingulum_Mid_L	0.653	63	SupraMarginal_L	0.912	93	Cerebelum_Crus2_L	0.352
4	Frontal_Sup_R	0.138	34	Cingulum_Mid_R	0.820	64	SupraMarginal_R	0.162	94	Cerebelum_Crus2_R	0.662
5	Frontal_Sup_Orb_L	0.052	35	Cingulum_Post_L	0.998	65	Angular_L	0.452	95	Cerebelum_3_L	0.010
6	Frontal_Sup_Orb_R	0.294	36	Cingulum_Post_R	0.146	66	Angular_R	0.414	96	Cerebelum_3_R	0.539
7	Frontal_Mid_L	0.365	37	Hippocampus_L	0.847	67	Precuneus_L	0.890	97	Cerebelum_4_5_L	0.653
8	Frontal_Mid_R	0.333	38	Hippocampus_R	0.389	68	Precuneus_R	0.396	98	Cerebelum_4_5_R	0.412
9	Frontal_Mid_Orb_L	0.733	39	ParaHippocampal_L	0.052	69	Paracentral_Lobule_L	0.771	99	Cerebelum_6_L	0.425
10	Frontal_Mid_Orb_R	0.779	40	ParaHippocampal_R	0.455	70	Paracentral_Lobule_R	0.910	100	Cerebelum_6_R	0.868
11	Frontal_Inf_Oper_L	0.800	41	Amygdala_L	0.176	71	Caudate_L	0.012	101	Cerebelum_7b_L	0.044
12	Frontal_Inf_Oper_R	0.470	42	Amygdala_R	0.386	72	Caudate_R	0.279	102	Cerebelum_7b_R	0.423
13	Frontal_Inf_Tri_L	0.300	43	Calcarine_L	0.490	73	Putamen_L	0.143	103	Cerebelum_8_L	0.951
14	Frontal_Inf_Tri_R	0.417	44	Calcarine_R	0.714	74	Putamen_R	0.008	104	Cerebelum_8_R	0.900
15	Frontal_Inf_Orb_L	0.283	45	Cuneus_L	0.732	75	Pallidum_L	0.646	105	Cerebelum_9_L	0.836
16	Frontal_Inf_Orb_R	0.973	46	Cuneus_R	0.750	76	Pallidum_R	0.561	106	Cerebelum_9_R	0.096
17	Rolandic_Oper_L	0.075	47	Lingual_L	0.685	77	Thalamus_L	0.990	107	Cerebelum_10_L	0.903
18	Rolandic_Oper_R	0.131	48	Lingual_R	0.256	78	Thalamus_R	0.594	108	Cerebelum_10_R	0.836
19	Supp_Motor_Area_L	0.698	49	Occipital_Sup_L	0.615	79	Heschl_L	0.095	109	Vermis_1_2	0.649
20	Supp_Motor_Area_R	0.473	50	Occipital_Sup_R	0.608	80	Heschl_R	0.160	110	Vermis_3	0.329
21	Olfactory_L	0.982	51	Occipital_Mid_L	0.514	81	Temporal_Sup_L	0.045	111	Vermis_4_5	0.762
22	Olfactory_R	0.913	52	Occipital_Mid_R	0.090	82	Temporal_Sup_R	0.830	112	Vermis_6	0.772
23	Frontal_Sup_Medial_L	0.340	53	Occipital_Inf_L	0.487	83	Temporal_Pole_Sup_L	0.070	113	Vermis_7	0.738
24	Frontal_Sup_Medial_R	0.183	54	Occipital_Inf_R	0.282	84	Temporal_Pole_Sup_R	0.917	114	Vermis_8	0.867
25	Frontal_Med_Orb_L	0.928	55	Fusiform_L	0.749	85	Temporal_Mid_L	0.900	115	Vermis_9	0.592
26	Frontal_Med_Orb_R	0.769	56	Fusiform_R	0.938	86	Temporal_Mid_R	0.113	116	Vermis_10	0.272
27	Rectus_L	0.096	57	Postcentral_L	0.878	87	Temporal_Pole_Mid_L	0.364			
28	Rectus_R	0.871	58	Postcentral_R	0.108	88	Temporal_Pole_Mid_R	0.860			
29	Insula_L	0.075	59	Parietal_Sup_L	0.984	89	Temporal_Inf_L	0.566			
30	Insula_R	0.744	60	Parietal_Sup_R	0.144	90	Temporal_Inf_R	0.343			
